# All-Small-Molecule Organic Solar Cells Based on a Fluorinated Small Molecule Donor With High Open-Circuit Voltage of 1.07 V

**DOI:** 10.3389/fchem.2020.00329

**Published:** 2020-04-28

**Authors:** Chunyan Liu, Nailiang Qiu, Yanna Sun, Xin Ke, Hongtao Zhang, Chenxi Li, Xiangjian Wan, Yongsheng Chen

**Affiliations:** ^1^Department of Chemistry and Chemical Engineering, Jining University, Qufu, China; ^2^The Centre of Nanoscale Science and Technology and Key Laboratory of Functional Polymer Materials, Renewable Energy Conversion and Storage Center (RECAST), College of Chemistry, Nankai University, Tianjin, China

**Keywords:** all-small-molecule organic solar cell, small molecule donor, small molecule acceptor, high open-circuit voltage, energy level control

## Abstract

A new small molecule donor with an acceptor-donor-acceptor (A-D-A) structure, namely DRTB-FT, has been designed and synthesized for all-small-molecule organic solar cells (ASM-OSCs). By introducing fluorine atoms on the thienyl substituent of the central benzodithiophene unit, DRTB-FT shows a low-lying highest occupied molecular orbital (HOMO) energy level of −5.64 eV. Blending with an A-D-A type acceptor F-2Cl, DRTB-FT based ASM-OSCs gave a power conversion efficiency (PCE) of 7.66% with a high open-circuit voltage (*V*_oc_) of 1.070 V and a low energy loss of 0.47 eV. The results indicate that high *V*_oc_ of ASM-OSC devices can be obtained through careful donor molecular optimization.

## Introduction

With the advantages of flexibility, light weight, roll to roll production and etc., bulk heterojunction (BHJ) organic solar cells (OSCs) have drawn extensive attention (Heeger, 2010; Qiu et al., 2017; Zhao F. et al., 2017; Cheng et al., 2018; Sun Y. et al., 2019). Significant progress has been obtained in the past decade, benefiting from the efforts in new material design and device optimization (Zhang Q. et al., 2014; He et al., 2015; Kan et al., 2015; Li Y. et al., 2018). Recently, non-fullerene acceptors (NFAs), especially acceptor-donor-acceptor (A-D-A) type small molecule acceptors, have demonstrated great success in OSCs due to the merits of facile synthesis, controllable absorption and fine-tuned energy levels (Lin et al., 2016; Zhao W. et al., 2017; Li T. et al., 2018; Zhang et al., 2018; Cui et al., 2019; Yuan et al., 2019).

From the perspective of donor materials, NFA based OSCs consist of polymer solar cells and small-molecule solar cells. Using polymer donor materials, both single junction and tandem OSCs have achieved the impressive power conversion efficiencies (PCEs) of 15–18% (Meng et al., 2018; An et al., 2019; Fan et al., 2019; Jiang et al., 2019; Lin et al., 2019; Sun H. et al., 2019; Wu Y. et al., 2019; Xiong et al., 2019; Yan et al., 2019). So far, all-small-molecule OSCs (ASM-OSCs), i.e., devices based on both small molecule donor and acceptor, have shown relatively lower performance compared with that of polymer donor based devices (Zhang Q. et al., 2014; Kan et al., 2015; Badgujar et al., 2016; Yang et al., 2017; Privado et al., 2018; Wang et al., 2018a; Duan et al., 2019). Nevertheless, with the advantages of versatile molecules design, less variation among different batches and thus easiness of property control for small molecules, ASM-OSCs are strongly believed to have the potential to achieve and even surpass the performance of polymer donor counterparts (Chen et al., 2013). To date, with the extensive study of small molecular design and device optimization for ASM-OSCs, the PCEs over 10% have been achieved (Bin et al., 2018; Wang et al., 2018a,b; Chen et al., 2019; Cheng et al., 2019; Gao et al., 2019; Ge et al., 2019; Wu H. et al., 2019; Yue et al., 2019; Zhou et al., 2019). Notably, a PCE over 14% has been obtained just recently by Wei et al. indicating the great potential of ASM-OSCs (Zhou et al., 2019).

**Figure 1:**
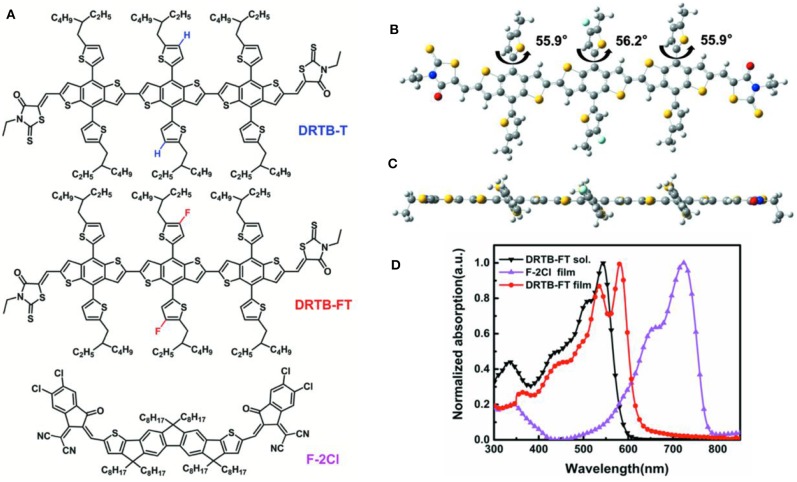
**(A)** Chemical structures of DRTB-T, DRTB-FT, and F-2Cl; **(B)** Top view and **(C)** Side view of the optimized molecular geometries of DRTB-FT at B3LYP/6-31G*; **(D)** Absorption spectra of DRTB-FT and F-2Cl.

Presently, compared with the comprehensive study of small molecule NFA, less attention has been paid to small molecule donors, which in fact play a vital role same as acceptors in OSCs. Among many properties that should be considered for designing active layer materials, matched energy levels between donor and acceptor not only contribute to charge transfer and exciton separation, but also have great influence on the open-circuit voltage (*V*_oc_) of OSC devices (Tang et al., 2017, 2019). Therefore, it is important to efficiently tune energy levels for designing active layer molecules. Among various strategies for tuning the energy level, introducing fluorine atoms into the designing molecule has been proved to be a simple way to downshift the frontier molecular orbital energy level, which has been used successfully in molecular design (Tang et al., 2018; Chen et al., 2019; Ge et al., 2019; Wu H. et al., 2019; Yue et al., 2019), e.g., PM6, a well-known polymer donor synthesized by Hou's group (Li W. et al., 2018). Recently, we have reported an ASM-OSC based on a non-fluorinated donor DRTB-T and an acceptor F-2Cl, showing a PCE of 10.76%, but a relatively lower *V*_oc_ of 0.969 V compared with other ASM-OSCs (Wang et al., 2018b). With these considerations, we have designed and synthesized a new small molecule donor with an A-D-A structure, namely DRTB-FT (as shown in Figure 1A), in which fluorine atoms are introduced on the thienyl substituent of central benzodithiophene (BDT) unit (Zhang and Li, 2014). As expected, DRTB-FT shows a lower highest occupied molecular orbital (HOMO) energy level of −5.64 eV in contrast to DRTB-T (−5.51 eV) (Yang et al., 2017). Notably, DRTB-FT has nearly the same solid film absorption ranging from 300 to 600 nm compared with that of DRTB-T, which is well complementary to that of an A-D-A type acceptor F-2Cl (500–800 nm). Thus, ASM-OSC based on DRTB-FT:F-2Cl gave a PCE of 7.66% with a high *V*_oc_ of 1.070 V and a low energy loss (*E*_loss_) of 0.47 eV. The results indicate that high *V*_oc_ of ASM-OSC device can be obtained through careful donor molecular optimization. The moderate performance was mainly attributed to the low FF (0.532) and *J*_sc_ (-13.46 mA cm^−2^) owing to the unmatched charge carrier mobilities and unfavorable active layer morphology.

## Experimental Section

### Materials and Synthesis

**Scheme 1:**
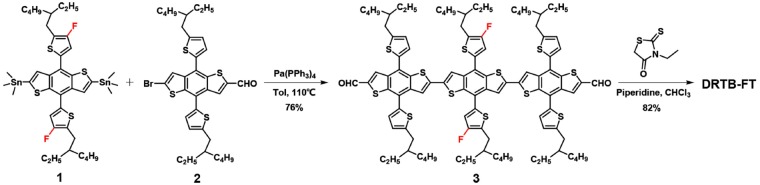
Synthetic route of DRTB-FT.

DRTB-FT was synthesized following the procedure in Scheme 1. F-2Cl was prepared according to the reported procedure (Wang et al., 2018b). The materials were commercially available and used without further purification. All manipulations and reactions were carried out following the standard Schlenk technique under argon protection.

**Compound 3**. Under argon protection, the weighed compound 1 (0.40 g, 0.425 mmol) and 2 (0.64 g, 0.936 mmol) were dissolved in toluene(15 mL). Subsequently, Pd(PPh_3_)_4_ (50 mg) was added as catalyst. The solution was stirred under dark condition, keeping the temperature at 110°C for 20 h. After removing the solvent, compound 3 was obtained as a red solid by silica gel chromatography (PE/CH_2_Cl_2_ = 1:2 as eluent) (0.59 g, 76%). ^1^H NMR (400 MHz, CDCl_3_), δ (ppm): 9.71 (s, 2H), 8.10 (s, 2H), 7.51 (s, 2H), 7.47 (s, 2H), 7.30 (d, 2H), 7.19 (d, 4H), 6.93 (d, 4H), 2.94–2.90 (m, 12H), 1.77-1.72 (m, 6H), 1.48–1.38 (m, 48H), 1.04–0.95 (m, 36H) (Figure S1). ^13^C NMR (100 MHz, CDCl_3_), δ(ppm): 183.74, 155.61, 153.11, 146.94, 146.47, 143.13, 141.48, 139.26, 138.90, 138.52, 137.77, 137.08, 135.78, 135.46, 133.95, 133.57, 133.48, 128.33, 126.07, 125.64, 125.51, 124.36, 123.06, 122.75, 122.57, 120.83, 118.14, 117.89, 41.35, 40.71, 34.38, 32.63, 32.59, 29.68, 29.53, 28.93, 25.86, 25.71, 23.06, 14.18, 10.87. MALDI-TOF MS: calcd for C_104_H_120_F_2_O_2_S_12_ [M^+^], 1824.84; found: 1824.77.

**Compound DRTB-FT**. Compound 3 (150 mg, 0.082 mmol) and 3-ethyl-rhodanine (133 mg, 0.82 mmol) were added in CHCl_3_ (20 mL). Under stirring the solution was deoxygenated using argon gas for 10 min. Subsequently, piperidine (0.1 mL) was added by syringe. After stirring at 60°C for 12 h, water (100 mL) was added and the product was extracted using CHCl_3_ (2 × 80 mL). The combined CHCl_3_ solution was washed twice using water and then dried by anhydrous Na_2_SO_4_. After removal of solvent, the crude product was chromatographied by silica gel (PE/CH_2_Cl_3_ = 1:3 as eluent), obtaining a powdery dark purple solid (142 mg, 82%). ^1^H NMR (400 MHz, CDCl_3_), δ (ppm): 7.66 (s, 2H), 7.43 (s, 2H), 7.34 (s, 2H), 7.30 (s, 2H), 7.26–7.23 (m, 4H), 7.16 (s, 2H), 6.94-6.92 (m, 4H), 4.02–4.00 (m, 4H), 2.96-2.87 (m, 12H), 1.81–1.78 (m, 6H), 1.57–1.42 (m, 48H), 1.19-1.16 (t, 6H), 1.06–0.92 (m, 36H). ^13^C NMR (100 MHz, CDCl_3_), δ (ppm): 189.92, 164.84, 153.75, 151.21, 144.84, 144.50, 139.73, 136.68, 136.43, 136.06, 135.62, 135.52, 134.92, 134.35, 133.98, 131.78, 129.46, 126.51, 126.25, 123.64, 123.28, 122.73, 121.82, 121.09, 120.87, 120.68, 120.50, 118.85, 118.69, 116.27, 115.99, 39.52, 39.43, 38.78, 37.74, 32.60, 32.54, 30.88, 30.77, 27.77, 27.14, 27.08, 26.99, 23.98, 23.91, 23.81, 23.17, 21.29, 21.25, 12.38, 12.31, 10.39, 9.15, 8.99 (Figure S2). MALDI-TOF MS: calcd for C_114_H_130_F_2_N_2_O_2_S_16_ [M^+^], 2111.30; found: 2110.65 (Figure S3).

### Measurements and Instruments

The ^1^H and ^13^C nuclear magnetic resonance (NMR) spectra were taken on by the Bruker AV400 Spectrometer. Matrix assisted laser desorption/ionization time-of-flight (MALDI-TOF) mass were measured using a Bruker Autoflex III instrument. X-ray diffraction (XRD) experiments were carried out with a Bruker Model D8 FOCUS X-ray diffractometer with Cu Kα radiation (λ = 1.5406 Å) at a current of 40 mA and a generator voltage of 40 kV. The UV-vis spectra were performed on a JASCO V-570 spectrophotometer. Cyclic voltammetry (CV) experiments were carried out using a LK98B II Microcomputer-based Electrochemical Analyzer and a conventional three-electrode configuration with a saturated calomel electrode (SCE) as the reference electrode, a Pt wire as the counter electrode, and a glassy carbon electrode as the working electrode at room temperature. The measurement was carried out employing acetonitrile solution of tetrabutylammonium phosphorus hexafluoride (*n*-Bu_4_NPF_6_, 0.1 M) as the supporting electrolyte with the scan rate of 100 mV s^−1^. The LUMO (lowest unoccupied molecular orbital) and HOMO values were obtained following the formula *E*_LUMO_ = – (4.80 + *E*_re_^onset^) and *E*_HOMO_ = – (4.80 + *E*_ox_^onset^), where *E*_re_^onset^ and *E*_ox_^onset^ can be estimated from the CV curves, respectively. The geometry structures of DRTB-FT was optimized through Density functional theory (DFT) calculations (B3LYP/6-31G^*^) according to Gaussian 16 (Lee et al., 1988; Frisch et al., 2016).

The current density-voltage (*J*-*V*) characteristics of ASM-OSC devices were measured with a Keithley 2400 source-measure unit. Using a SAN-EI XES-70S1 AAA class solar simulator, photocurrent was performed under illumination with simulated 100 mW cm^−2^ AM 1.5G irradiation. The external quantum efficiency (EQE) curve was obtained by a QE-R Solar Cell Spectral Response Measurement System.

Atomic force microscope (AFM) and Transmission electron microscopy (TEM) images were obtained on a Bruker MultiMode 8 in tapping mode and Philips Technical G2 F20 at 200 kV, respectively. Space charge limited current (SCLC) mobility was performed by a diode configuration of glass/ZnO/active layer/Al for electron mobility and ITO/PEDOT:PSS/active layer/Au for hole mobility, respectively. The results were fitted via a space charge limited form, being described as the equation:

$$\begin{array}{l} J=\frac{9\varepsilon_{0}\varepsilon_{r}\mu_{0}V^{2}}{8L^{3}}\text{exp}\left(0.89\beta \sqrt{\frac{V}{L}}\right) \end{array}$$

where *J* is the current density, ε_0_ is the permittivity of free space (8.85 × 10^−12^ F m^−1^), ε_r_ is the relative dielectric constant of the transport medium, μ_0_ is the hole or electron mobility, *V* (= *V*_appl_ – *V*_bi_) is the internal voltage in the device, where *V*_appl_ is the applied voltage to the device and V_bi_ is the built-in voltage due to the relative work function difference of the two electrodes, *L* is the film thickness of the active layer.

### Fabrication of OSC Devices

ASM-OSC devices were fabricated using a conventional structure of glass/ITO/PEDOT:PSS/Donor:Acceptor/PDINO/Al, in which the electronic transport material PDINO was designed by Zhang et al. (2014). As a common procedure: the cleaned ITO-coated glasses were treated by ultraviolet-ozone for 15 min and spin-coated with PEDOT:PSS solution at 4,800 rpm for 20 s. Subsequently, the glasses were baked at 150°C for 20 min and transferred into the glove box with argon gas. Chloroform solution of both donor and acceptor was spin-coated on the PEDOT:PSS at 1,700 rpm for 20 s. Afterward, PDINO was spin-coated on the active layer at 3,000 rpm for 20 s with the concentration of 1.0 mg/ml using EtOH as solvent. Finally, under the high vacuum, a cathode material Al was deposited for 50 nm onto PDINO layer. The work area of each device is about 0.04 cm^2^.

## Results and Discussion

### Materials Synthesis and Characterization

The synthesis of DRTB-FT is illustrated in Scheme 1. By Stille coupling reaction between compound 1 and 2, two commercially available BDT derivatives, compound 3 was synthesized. Subsequent Knoevenagel condensation reaction between compound 3 and 3-ethylrhodanine gave the final product in high yield. As shown in the experimental section above, DRTB-FT was characterized through NMR spectroscopy and MALDI-TOF MS. DRTB-FT exhibits good solubility in common organic solvents such as chlorobenzene, dichloromethane and chloroform. As shown in Figures 1B,C, the optimized geometry of DRTB-FT based on DFT (B3LYP/6-31G^*^) displays a good coplanar conformation. The structural ordering of pristine DRTB-FT and DRTB-T film was investigated by XRD analysis. According to the XRD results (Figure S4), a weak diffraction peak (100) at 4.44 (2θ) was observed for the DRTB-T film. In contrast, DRTB-FT film exhibits a strong diffraction peak (100) at 4.46 (2θ), indicating stronger stacking and crystallinity than DRTB-T.

### Optical and Electrochemical Properties

The UV-vis absorption spectra of DRTB-FT obtained in chloroform solution and in solid state are shown in Figure 1D. In chloroform solution, DRTB-FT exhibits an absorption maximum at 547 nm, and it shows a bathochromic-shifted and broadened absorption in the solid state ranging from 300 to 630 nm, which is well complementary to that of F-2Cl film. The optical bandgap (*E*_g_^opt^) obtained from DRTB-FT film absorption onset (622 nm) is 1.99 eV. Moreover, a stronger shoulder peak was observed in film absorption spectrum in comparison to the non-fluorinated counterpart DRTB-T, which reveals the stronger intermolecular stacking and π-π interactions in DRTB-FT solid film. The electrochemical behavior of DRTB-FT was investigated by CV experiments. As shown in Figure S5, the estimated reduction and oxidation potentials of DRTB-FT are −1.17V and 0.84V (vs Fc/Fc^+^), respectively. Therefore, the first oxidation event of DRTB-FT occurs at a higher oxidation potential compared with DRTB-T, leading to a deeper HOMO of −5.64 eV, which is in favor of obtaining a higher *V*_oc_ in the devices when combined with appropriate acceptor. The optical and electrochemical data of DRTB-FT and DRTB-T are listed in Table 1.

**Table 1:** Optical and electrochemical data of DRTB-T and DRTB-FT.

**Molecules**	**UV-vis**			**CV**		
	**λ_max_^sol^ (nm)**	**λ_max_^film^**	***E*_g_opt (eV) *^a^***	**HOMO (eV)**	**LUMO (eV)**	***E*_g_^cv^ (eV)**
DRTB-T^b^	545	545, 585	2.00	−5.51	−3.34	2.17
DRTB-FT	547	544, 587	1.99	−5.64	−3.61	2.03

a Optical bandgap was obtained from the onset wavelength of the film.

b *Yang et al. (2017)*.

### Photovoltaic Properties

**Figure 2:**
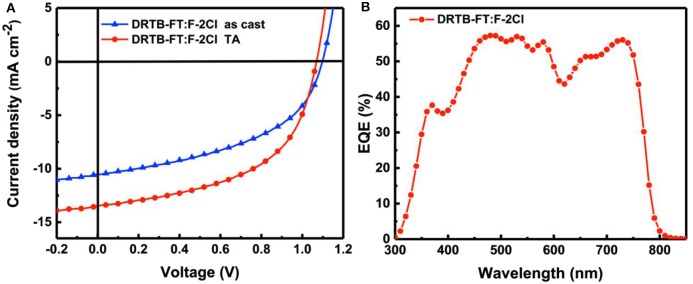
**(A)** Current density-voltage (*J*-*V*) curves of BHJ devices and **(B)** EQE curves of the optimal BHJ device based on DRTB-FT:F-2Cl.

ASM-OSC devices with a conventional structure of ITO/PEDOT:PSS/active layer/PDINO/Al were fabricated. The devices performance were optimized by varying the donor to acceptor weight ratio (D/A, w/w) in the active layer, applying solvent vapor annealing (SVA) and thermal annealing (TA) treatment and etc. The detailed device parameters are provided in Tables S1–S3. The photovoltaic parameters of the as-cast and optimal devices are listed in Table 2 and the corresponding *J*-*V* characteristics are shown in Figure 2A. For the as-cast device, a PCE of 5.72% with a high *V*_oc_ of 1.10 V was obtained. An improved photovoltaic performance with a PCE of 7.66% and a high *V*_oc_ of 1.07 V was achieved after TA treatment at 100°C for 10 min, and the corresponding *E*_loss_ is as low as 0.47 eV calculated via the equation *E*_loss_ = *E*_g_^opt^-e*V*_oc_, where *E*_g_^opt^ is the optical bandgap of F-2Cl film (1.54 eV). As expected, OSCs based on DRTB-FT:F-2Cl demonstrated a high *V*_oc_ owing to the low-lying HOMO energy level of the DRTB-FT donor. In contrast, the DRTB-T:F-2Cl based device has a *V*_oc_ of 0.969 V. However, limited by the low *J*_sc_ (13.46 mA cm^−2^) and FF (0.532), the optimized device based on DRTB-FT:F-2Cl gave a moderate PCE (7.66%). The unsatisfactory device performance can be ascribed to the unfavorable active layer morphology and unmatched charge carrier mobilities, as will be discussed in the following section.

**Table 2:** Photovoltaic parameters of OSCs based on DRTB-FT:F-2Cl and DRTB-T:F-2Cl.

**Donor**	**Treatment**	***V*_**oc**_ (V)**	***J*_**sc**_ (mA cm^**−2**^)**	**FF**	**PCE (%)**
DRTB-FT	As cast	1.098	10.77	0.478	5.65 (5.51)^a^
	TA	1.070	13.46	0.532	7.66 (7.45)^a^
DRTB-T	SVA	0.969	17.24	0.64	10.76^b^

a Average PCE values obtained from 20 devices are shown in parentheses;

b *Wang et al. (2018b)*.

The EQE spectrum of the optimal device is presented in Figure 2B, which exhibits a broad photo-to-current response from 300 to 790 nm. However, the maximum efficiency is only 58% at 471 nm, indicating a lower photoelectron conversion process in comparison to that of DRTB-T based device (Wang et al., 2018b). The calculated *J*_sc_ value obtained from the integration of the EQE curve is 13.01 mA cm^−2^, which is in good agreement with the *J*_sc_ value from the corresponding *J-V* curve with about 3% mismatch.

**Figure 3:**
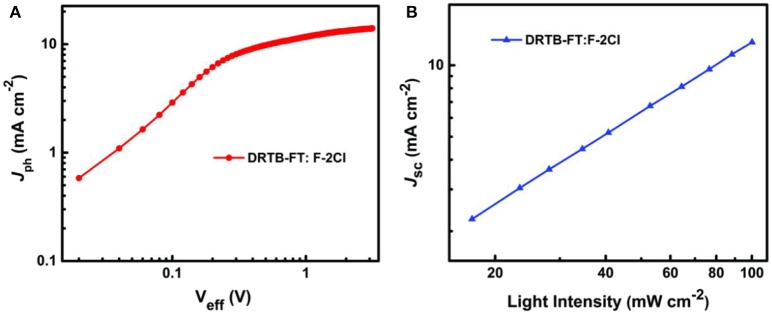
**(A)** Photocurrent density vs. effective voltage (*J*_ph_-*V*_eff_) and **(B)** double logarithmic plots of *J*_sc_ as a function of incident light intensity for the optimal device based on DRTB-FT:F-2Cl.

To explore the reason for lower *J*_sc_ and FF of the ASM-OSC, the exciton dissociation and extraction process in the active layer of optimal device was investigated. Figure 3A shows the relation between photocurrent density *J*_ph_ (*J*_ph_ = *J*_L_ – *J*_D_) and effective voltage *V*_eff_ (*V*_eff_ = *V*_0_ – *V*_a_), where *J*_L_, *J*_D_, *V*_0_, and *V*_a_ are the light current density, the dark current density, the voltage at *J*_ph_ = 0 and the applied voltage, respectively. It can be seen that the *J*_ph_ curve of DRTB-FT:F-2Cl based device is hard to reach the saturation current density (*J*_sat_) even at a high *V*_eff_. Lower *J*_ph_/*J*_sat_ values of 85.9% and 61.0% were obtained under the short-circuit and maximal output power conditions, respectively, which demonstrated a poor exciton dissociation and charge extraction processes and limited *J*_sc_ and FF. For further study, the bimolecular recombination degree in active layer was estimated on the basis of the formula of *J*_sc_ ∝ *P*^α^, where α is the exponential factor. When α value was closer to 1, the less bimolecular recombination in blend film. The fitted slope (α) is calculated to be 0.964 for the devices based on DRTB-FT as shown in Figure 3B, which is relatively lower compared with that of DRTB-T based device (0.985) (Wang et al., 2018b), indicating more bimolecular recombination in DRTB-FT:F-2Cl active layer.

**Figure 4:**
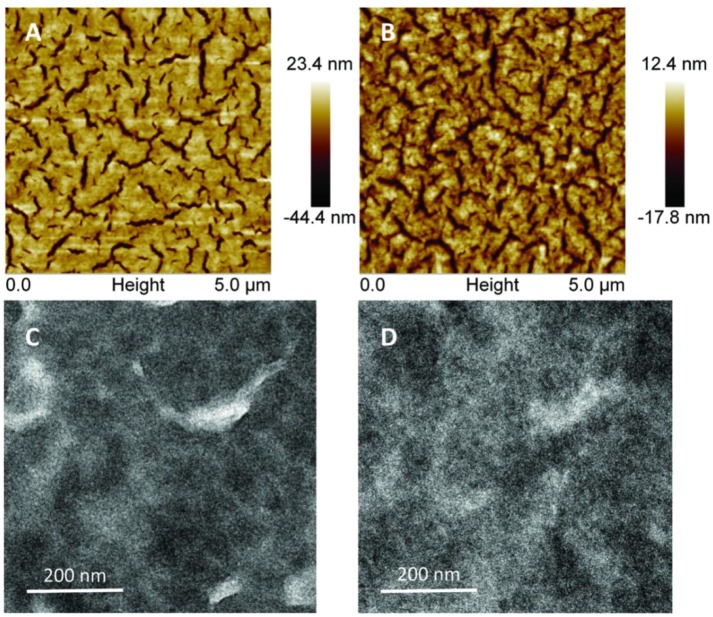
Tapping-mode AFM height images of the active layers of the **(A)** as-cast and **(B)** optimal DRTB-FT:F-2Cl blend films. TEM images of the **(C)** as-cast and **(D)** optimal DRTB-FT:F-2Cl blend films.

The influence of active layer morphology on device performance was investigated by AFM and TEM technology. Figure 4A shows that the surface topography of the as-cast DRTB-FT:F-2Cl blend film is relatively rough in terms of the large root-mean-square (RMS) roughness (9.2 nm), which reveals the existence of excessive aggregation in the as-cast film due to the strong stacking and crystallinity of donor molecules. For the optimal blend film (Figure 4B), a relatively smaller RMS roughness of 4.4 nm was observed, which is beneficial to form better contact between the film and the electrode, and thus to promote charge extraction. TEM images are in consistent with the AFM, from Figure 4C, it can be seen that an oversized phase separation and more defects in the as-cast blend film, and the morphology becomes better obviously after TA treatment (Figure 4D). However, there was still no clear fiber structure or suitable phase separation in the optimized blend film, which might account for the low *J*_sc_ and FF.

To investigate the charge-transporting behavior of the studied devices, the electron and hole mobilities (μ_*e*_ and μ_h_) were measured by the SCLC method as described in experimental section. According to Figure S6, the electron mobility of μ_*e*_=2.11 × 10^−4^ cm^−2^V^−1^s^−1^ and hole mobility of μ_h_ = 4.51 × 10^−5^ cm^−2^V^−1^s^−1^ were obtained for the as-cast device. After TA treatment, the charge mobilities were raised to μ_*e*_= 4.12 × 10^−4^ cm^−2^V^−1^s^−1^ and μ_h_ = 8.56 × 10^−5^ cm^−2^V^−1^s^−1^, respectively. The relatively lower and unbalanced hole and electron mobilities could lead to more charge recombination, thus the lower FF and *J*_sc_.

## Conclusion

In summary, we have designed and synthesized a new small molecule and A-D-A type donor named DRTB-FT, taking the fluorothienyl-substituted benzodithiophene as the central unit. The introduction of fluorine atoms enables DRTB-FT to get a low HOMO of −5.64 eV. When combined with an acceptor F-2Cl, the optimal ASM-OSC device gave a PCE of 7.66%, especially a high *V*_oc_ of 1.070 V and a low *E*_loss_ of 0.47 eV. The results indicate that high *V*_oc_ of ASM-OSCs can be obtained through careful donor molecular optimization. On the other hand, morphology control also plays a critical role in ASM-OSCs. In this case, the low *J*_sc_ and FF are caused by the unfavorable active layer morphology with large aggregation of small molecule donors. It is believed that high performance ASM-OSCs will be achieved through synergistic study of molecular design with complementary absorption, matched energy levels and morphology control.

## Data Availability Statement

All datasets generated for this study are included in the article/Supplementary Material.

## Author Contributions

CLiu synthesized the DRTB-FT and measured the optical-electric properties of DRTB-T and DRTB-FT. NQ fabricated and optimized the devices, and wrote the manuscript. YS contributed to the morphology characterization. XK ran the calculations of DRTB-FT, HZ, CLi, XW, and YC conceived and directed the project. All authors contributed to the whole work.

## Conflict of Interest

The authors declare that the research was conducted in the absence of any commercial or financial relationships that could be construed as a potential conflict of interest.
