# Supplementary material for: All-Small-Molecule Organic Solar Cells Based on a Fluorinated Small Molecule Donor With High Open-Circuit Voltage of 1.07 V
Source: Front Chem. 2020 Apr 28;8:329. doi: 10.3389/fchem.2020.00329 (PMC7198867; doi:10.3389/fchem.2020.00329)
Supplement: Supplementary file 1 [file Table_1.docx]

Supplementary Material

All-small-molecule organic solar cells based on a fluorinated small molecule donor with high open-circuit voltage of 1.07 V

**Chunyan Liu, Nailiang Qiu^*^, Yanna Sun, Xin Ke, Hongtao Zhang, Chenxi Li, Xiangjian Wan^*^, Yongsheng Chen**

**Table S1** Photovoltaic performance of the solar cells based on DRTB-FT:F-2Cl blend films with different Donor:Acceptor ratio under illumination of AM 1.5 G, 100 mW cm^-2^.

| D:A (w/w) | *V*_oc_ (V) | *J*_sc_ (mA cm^-2^) | FF | PCE (%)*^a^* |
| --- | --- | --- | --- | --- |
| 1:0.6 | 1.086 ± 0.010 | 9.60 ± 0.23 | 0.465 ± 0.011 | 4.96 (4.85) |
| 1:0.8 | 1.098 ± 0.007 | 10.52 ± 0.25 | 0.477 ± 0.009 | 5.65 (5.51) |
| 1:1 | 1.092 ± 0.011 | 10.23 ± 0.41 | 0.448 ± 0.012 | 5.38 (5.00) |

*^a^* The values in the parentheses are the average PCEs from 20 devices.

**Table S2** Photovoltaic performance of the solar cells based on DRTB-FT:F-2Cl (1:0.8, w/w) blend films with different TA temperature under illumination of AM 1.5 G, 100 mW cm^-2^.

| Temperature ( ℃ ) | *V*_oc_ (V) | *J*_sc_ (mA cm^-2^) | FF | PCE (%)*^a^* |
| --- | --- | --- | --- | --- |
| 80 | 1.085 ± 0.008 | 12.73 ± 0.19 | 0.496 ± 0.009 | 7.01 (6.85) |
| 100 | 1.064 ± 0.013 | 13.21 ± 0.25 | 0.530 ± 0.010 | 7.66 (7.45) |
| 120 | 1.050 ± 0.010 | 13.37 ± 0.26 | 0.501 ± 0.012 | 7.22 (7.03) |

*^a^* The values in the parentheses are the average PCEs from 20 devices.

**Table S3** Photovoltaic performance of the solar cells based on DRTB-FT:F-2Cl (1:0.8, w/w) blend films with different SVA time under illumination of AM 1.5 G, 100 mW cm^-2^.

| Time ( s ) | *V*_oc_ (V) | *J*_sc_ (mA cm^-2^) | FF | PCE (%)*^a^* |
| --- | --- | --- | --- | --- |
| 40 | 1.087 ± 0.013 | 10.85 ± 0.24 | 0.480 ± 0.011 | 5.84 (5.66) |
| 60 | 1.076 ± 0.011 | 12.01 ± 0.17 | 0.506 ± 0.008 | 6.79 (6.54) |
| 80 | 1.087 ± 0.009 | 11.52 ± 0.22 | 0.496 ± 0.010 | 6.42 (6.21) |

*^a^* The values in the parentheses are the average PCEs from 20 devices.


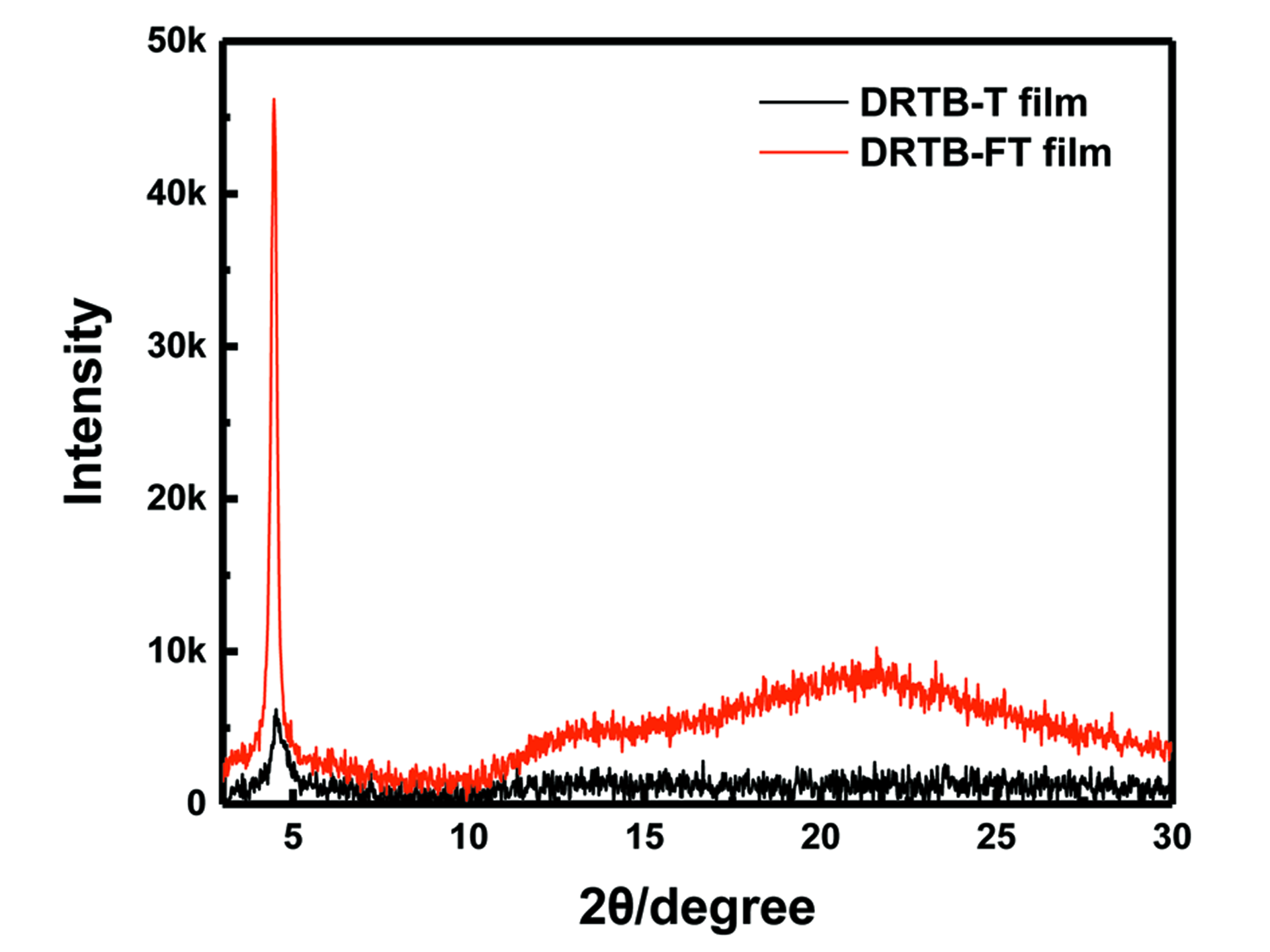


**Figure S1** XRD patterns of DRTB-T and DRTB-FT films with thermal annealing at 100 ^0^C for 10 min.





**Figure S2** Cyclic voltammogram of DRTB-FT in acetonitrile solution with 0.1 mol L^-1^ n-Bu_4_NPF_6_ at a scan rate of 100 mV s^-1^.

**
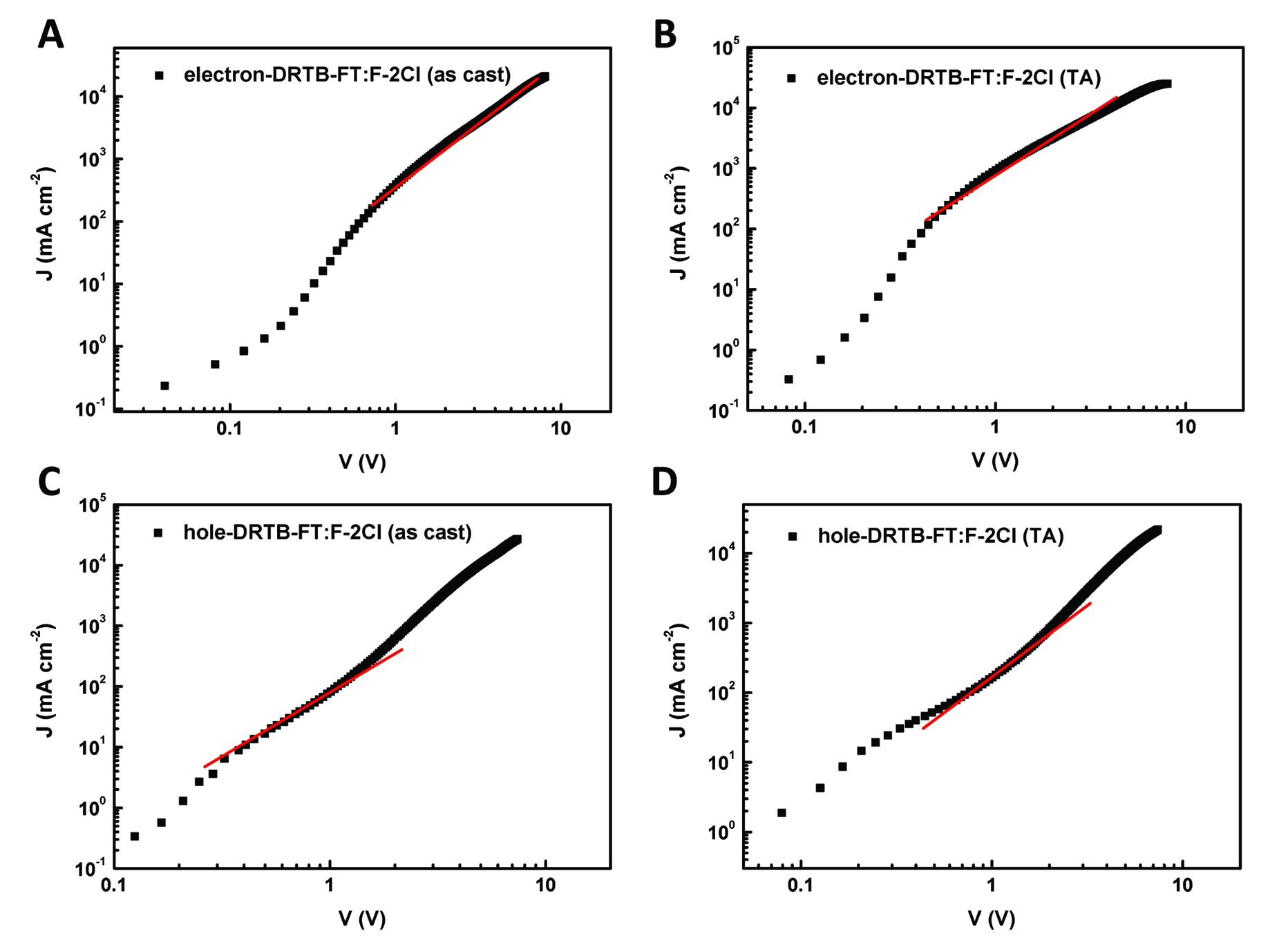
**

**Figure S3** The current-density-voltage (*J-V*) plots for electron-only devices based on DRTB-FT:F-2Cl with (A) as cast and (B) thermal annealing, and hole-only devices based on DRTB-FT:F-2Cl with (C) as cast and (D) thermal annealing.


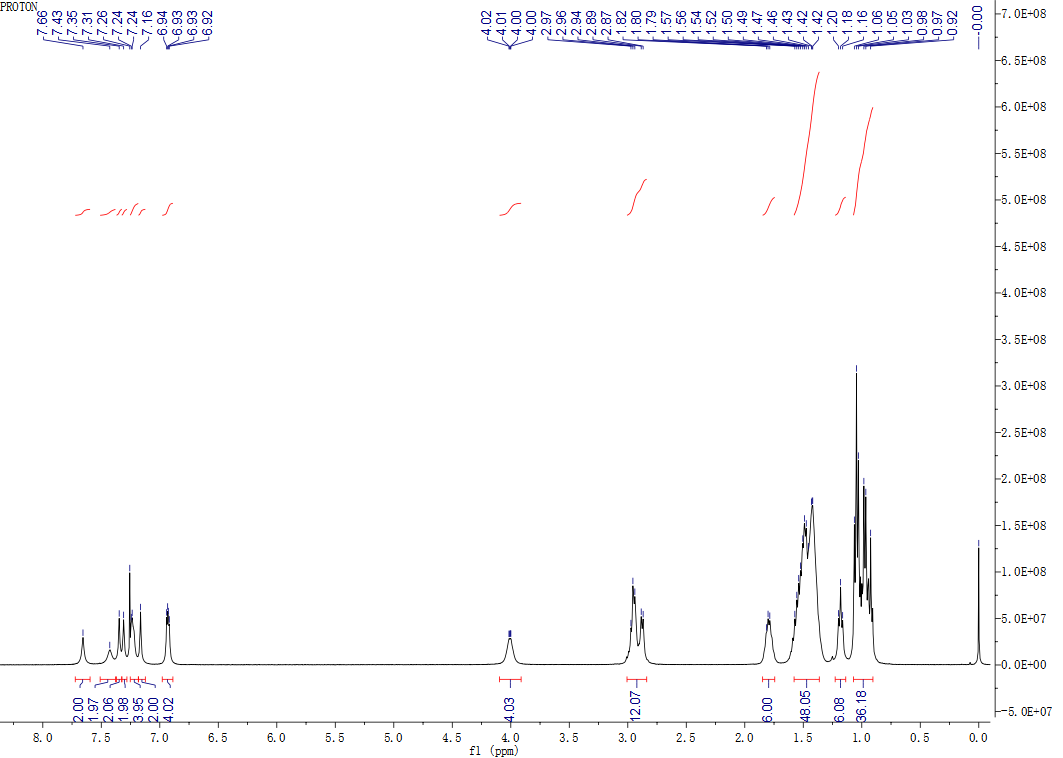


**Figure S4** ^1^H NMR spectra of compound DRTB-FT at 300K in CDCl_3_.


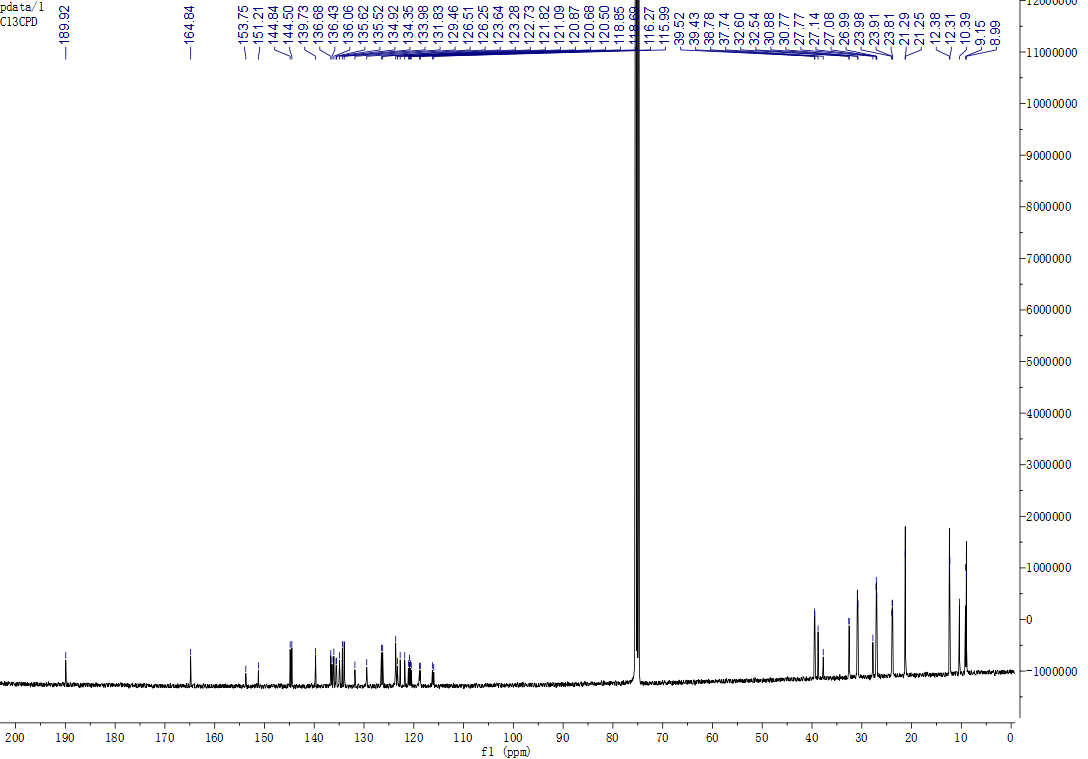


**Figure S5** ^13^C NMR spectra of compound DRTB-FT at 300K in CDCl_3_.

**Figure S6** The MALDI-TOF MS plot of compound DRTB-FT.
